# A Combined Use of Rhizobacteria and Moringa Leaf Extract Mitigates the Adverse Effects of Drought Stress in Wheat (*Triticum aestivum* L.)

**DOI:** 10.3389/fmicb.2022.813415

**Published:** 2022-06-21

**Authors:** Irfana Lalarukh, Sami A. Al-Dhumri, Laith Khalil Tawfeeq Al-Ani, Rashid Hussain, Khalid Awadh Al Mutairi, Nida Mansoora, Syeda Fasiha Amjad, Mohamed H. H. Abbas, Ahmed A. Abdelhafez, Peter Poczai, Khem Raj Meena, Tarek M. Galal

**Affiliations:** ^1^Department of Botany, Government College Women University, Faisalabad, Pakistan; ^2^Department of Biology, Al Khumra University College, Taif University, Taif, Saudi Arabia; ^3^Department of Plant Protection, College of Agriculture Engineering Science, University of Baghdad, Baghdad, Iraq; ^4^School of Biology Science, Universiti Sains Malaysia, George Town, Malaysia; ^5^Department of Horticultural Sciences, Faculty of Agriculture and Environment, The Islamia University of Bahawalpur, Bahawalpur, Pakistan; ^6^Department of Biology, Faculty of Science, University of Tabuk, Tabuk, Saudi Arabia; ^7^Department of Botany, University of Agriculture Faisalabad, Faisalabad, Pakistan; ^8^Department of Soils and Water, Faculty of Agriculture, Benha University, Benha, Egypt; ^9^Department of Soils and Water, Faculty of Agriculture, New Valley University, Kharga, Egypt; ^10^National Committee of Soils Science, Academy of Scientific Research and Technology, Cairo, Egypt; ^11^Botany Unit, Finnish Museum of Natural History, University of Helsinki, Helsinki, Finland; ^12^Department of Microbiology, College of Basic Sciences and Humanities, Dr. Rajendra Prasad Central Agricultural University, Pusa, India; ^13^Department of Botany and Microbiology, Faculty of Science, Helwan University, Cairo, Egypt; ^14^Department of Biology, College of Sciences, Taif University, Taif, Saudi Arabia

**Keywords:** PGPR, agroecological zones, drought stress, *Pseudomonas*, nitrogen-fixing bacteria, biofertilizers, bioinnoculants

## Abstract

Less nutrient availability and drought stress are some serious concerns of agriculture. Both biotic and abiotic stress factors have the potential to limit crop productivity. However, several organic extracts obtained from moringa leaves may induce immunity in plants under nutritional and drought stress for increasing their survival. Additionally, some rhizobacterial strains have the ability to enhance root growth for better nutrient and water uptake in stress conditions. To cover the knowledge gap on the interactive effects of beneficial rhizobacteria and moringa leaf extracts (MLEs), this study was conducted. The aim of this experimental study was to investigate the effectiveness of sole and combined use of rhizobacteria and MLEs against nutritional and drought stress in wheat. Nitrogen-fixing bacteria *Pseudomonas aeruginosa* (Pa) (10^8^ CFU ml^–1^) was inoculated to wheat plants with and without foliar-applied MLEs at two different concentrations (MLE 1 = 1:15 v/v and MLE 2 = 1:30 v/v) twice at 25 and 35 days after seed sowing (50 ml per plant) after the establishment of drought stress. Results revealed that Pa + MLE 2 significantly increased fresh weight (FW), dry weight (DW), lengths of roots and shoot and photosynthetic contents of wheat. A significant enhancement in total soluble sugars, total soluble proteins, calcium, potassium, phosphate, and nitrate contents validated the efficacious effect of Pa + MLE 2 over control-treated plants. Significant decrease in sodium, proline, glycine betaine, electrolyte leakage, malondialdehyde, hydrogen peroxide, superoxide dismutase (SOD), and peroxide (POD) concentrations in wheat cultivated under drought stress conditions also represents the imperative role of Pa + MLE 2 over control. In conclusion, Pa + MLE 2 can alleviate nutritional stress and drought effects in wheat. More research in this field is required to proclaim Pa + MLE 2 as the most effective amendment against drought stress in distinct agroecological zones, different soil types, and contrasting wheat cultivars worldwide.

## Introduction

Crop production is always under pressure to increase and sustain the food demands, considering the estimated increase in global population that might increase from the current 7.7 billion to approximately 9.6 billion in the year 2050 (Zulfiqar et al., 2020). Moreover, constantly changing climatic conditions are the other major challenge for sustainable crop production (Wheeler and Von Braun, 2013). Several abiotic and biotic stress factors such as temperature, water-logging, salinity, drought, weed, and pest infestations critically limit crop production (Parajuli et al., 2019). Climate change leads to widely drought-affected areas, such as tropics (Grover et al., 2011).

Water constitutes approximately 80–90% of the total biomass of herbaceous plants and is crucial in almost all plant physiological processes, a principal means of nutrients and metabolite transport (Lisar et al., 2012). Water scarcity is one of the leading plant stress factors. A drought effect reduces cell turgor and water potential causing adversities in carrying out plant’s normal physiological functions (Lisar et al., 2012). In fact, water is the key determining factor for crop growth and productivity, thus playing an essential role in species distribution and evolution (Ngumbi and Kloepper, 2016).

The induction of drought stress tolerance has been described by several physiological and biochemical changes (Shukla et al., 2012). In spite of these tolerance strategies including elaborated antioxidative activities, losses to crop production are increasing rapidly. Chemical fertilizers are largely being used to increase agricultural production, which is a matter of concern due to their improper use and potential adverse effects on human health and environment (Ruzzi and Aroca, 2015; Jiménez-Gómez et al., 2018). Expensive agrochemicals and synthetic fertilizers are commercially undesirable (Colla et al., 2015). Hence, to maintain and increase the yield, it is crucial to devise some efficient, low-cost, less time taking, and environment-friendly techniques to cope with drought conditions and achieve a sustainable agricultural system (Venkateswarlu and Shanker, 2009; Zulfiqar et al., 2019).

Several microorganisms, mainly bacteria, colonize the plant root zone (Kaushal and Wani, 2016; Barnawal et al., 2019). The beneficial associations between microbes and roots are an important determinant of soil texture, water conservation, and plant health. Plant growth-promoting rhizobacteria (PGPR) improve nutrient uptake by roots, growth, and consequently crop yield by causing biochemical changes in the plant body, meanwhile protecting from a variety of other plant diseases (Khan et al., 2020; Singh et al., 2021; Soumare et al., 2021). Furthermore, improvement in soil texture and fertility enhances crop production (Nardi et al., 2009).

Moringa leaf extract (MLE), as a plant biostimulant in the foliar application, enhances the growth of plants that are grown even under abiotic stress conditions (Semida and Rady, 2014). *Moringa oleifera* Lam belongs to the Moringaceae family and native to the subcontinent (Shahzad et al., 2013). Moringa leaves are rich in both micronutrients and macronutrients, i.e., N, P, Ca, K, Na, Zn, Mg, B, Cu, Mn, and Fe (Yasmeen et al., 2014). These mineral nutrients can supplement the nutritional demands of stressed grown crops and help in decreasing the use of agrochemicals and synthetic fertilizers (Zulfiqar et al., 2020). Spraying MLE diluted solution on plant leaves seems to have a considerable positive impact, i.e., delay in senescence, higher sugar contents, increased plant height, and bigger fruits and seeds (Yasmeen et al., 2013a; Nasir et al., 2014). This validates the potential effects of MLE to be used to improve plant vigor specifically in suboptimal environmental situations like drought stress as a foliar application. MLE foliar spray increased approximately 20–35% yield of several plants (Yasmeen et al., 2012).

Wheat (*Triticum aestivum* L.) is one of the most important cereal crops worldwide, considering its production and human consumption. Wheat supplements almost one-third of the total global population. Harvesting more yields to meet the future food demands of the increasing population is a major agricultural concern at all times. Different environmental factors contribute to crop yields (Yasmeen et al., 2012; Fasiha Amjad et al., 2021). Wheat in Pakistan experiences severe abiotic constraints during all developmental stages (Rashid et al., 2018), and drought stress is the main limiting factor among abiotic stresses for wheat yield (Budak et al., 2013; Mutumba et al., 2018).

Previously, the effects of exogenous applied MLE and treating plants with nitrogen-fixing bacteria (NFB) were studied solely on wheat grown at high temperatures. This study covers the knowledge gap in the combined use of NFB and MLE under normal and heat stress situations. In earlier studies, treating plants with rhizobacteria and the effects of foliar sprayed MLE were assessed solely on wheat grown under drought stress conditions by considering their ameliorative implications on various physiological and biochemical attributes but their combined implications are still not confirmed.

Therefore, this study aimed to investigate the best treatment combination for alleviation of heat stress in wheat. Anaj 17 genotype was selected because it is one of the latest genotypes developed in the past 5 years in Pakistan and it performed well under abiotic stress conditions so we wanted to check its response to the application of MLE and rhizobacteria. It is hypothesized that the combined use of MLE and NFB might be a better approach than the sole application to improve wheat growth attributes under heat stress. This study was conducted to analyze the ameliorative and best co-application of applied amendments in alleviating the drought stress effects on wheat plants. It is hypothesized that MLE and rhizobacterial combined application might be an effective approach to improve wheat attributes than their sole applications in drought effects. In earlier studies, treating plants with rhizobacteria and the effects of foliar sprayed MLE were assessed solely on wheat grown under drought stress conditions by considering their ameliorative implications on various physiological and biochemical attributes but their combined implications are still not confirmed. Therefore, this study is conducted to analyze the ameliorative and best co-application of applied amendments in alleviating the drought stress effects on wheat plants. It is hypothesized that MLE and rhizobacterial combined application might be an effective approach to improve wheat attributes than their sole applications in drought effects.

## Materials and Methods

### Experimental Site and Design

The wheat crop was sown at the experimental site of the Government College University Faisalabad (30°–31.5° N and 73°–74° E, 184.4 m above sea level). A greenhouse pot experiment was carried out from November to January. The experimental layout was a randomized complete block design (RCBD) that was replicated three times.

### Soil Characteristics

The clay loam soil (8–12 inches in depth) collected from the experimental site of the Government College University Faisalabad was air-dried and sieved through a 2-mm sieve. The collected soils were sterilized through solarization, by covering the soil with a thin layer of plastic sheet. The heat from the sun builds up the temperature of the soil to kill most of the bacteria, weeds, and pests (Stapleton et al., 2008). Some soil chemical characteristics that were analyzed before the experiment include EC 7.78 (d Sm^–1^), pH (water) 7.3; organic matter contents 1.38%; available N 0.032 ppm, available P 5.93 ppm, and available K 32.3 ppm.

### Seed Collection and Sterilization

A well-adapted wheat genotype Anaj 17, which performs well under abiotic stress conditions, was selected. Seeds were disinfected using 95% ethanol and washed using 70% sodium hypochlorite solution followed by rinsing with distilled water three times.

### Treatments

The treatments (two sets of pots) were as follows: (i) control (untreated with foliar spray, no bacterial inoculation], (ii) *Pseudomonas aeruginosa* (Pa) (strain) [inoculation with Pa], (iii) MLE 1 [foliar sprayed MLE at 1:15], (iv) MLE 2 [foliar sprayed MLE at 1:30], (v) Pa + MLE 1 [inoculation with Pa bacteria + foliar sprayed MLE at 1:15], and (vi) Pa + MLE 2 [inoculation with Pa bacteria + foliar sprayed MLE at 1:30].

### Fertilizer and Seed Sowing

At the start of the experiment, the soil was fertilized with a basal dose of N–P–K fertilizer (0.51–0.45–0.38 N–P–K g) using urea (46% N), sulfate of potash (50% K_2_O), and diammonium phosphate (46% P_2_O_5_, 18% N) [24]. Initially, ten seeds were sown in each pot containing 12 kg clay loamy soil (25 cm diameter × 30 cm height), and after complete emergence, they were thinned to six plants per pot.

### Moringa Leaf Extract, Pa (Strain), and Establishment of Drought Stress

An aerobic PGPR strain of free-living soil nitrogen-fixing bacteria; Pa (Pa strain) isolated from the rhizosphere of wheat roots growing in local field areas by serial dilution method was used in this study. The serial dilutions up to 10^–7^ were spread on Luria-Bertani (LB) agar plates at 37°C temperature and inoculated overnight. Bacterial growth was determined by measuring optical density at 600 nm using a spectrophotometer (Gontia-Mishra et al., 2016). Before inoculation, the seeds were coated with 20% gum Arabic as an adhesive and immersed in a bacterial suspension of 10^8^ CFU ml^–1^ strength just before 15 min of sowing. After 20 days of seed sowing, one set of pots (half number) was shifted to canopies for the imposition of drought stress. Well-watered conditions were maintained at 70 FC (70% field capacity), while drought stress was maintained at 45 FC (45% field capacity). The plants that remained were kept at different water regimes until harvesting.

Fresh and disease-free moringa leaves were collected and rinsed with water. Notably, a 100-g leaf sample was extracted in 1 L distilled water (1:10 w/v) for 15 min (Yasmeen et al., 2013b). Later, MLE was filtered and diluted with distilled water at two concentrations: MLE 1 = 1:20 v/v and MLE 2 = 1:30 v/v. Some chemical properties of moringa leaves are total chlorophyll = 3.79 mg g^–1^ FW, carotenoids = 1.58 mg g^–1^ FW, total phenolics = 1.68 μmol g^–1^ FW, mg g^–1^ FW, nitrogen = 14.13 mg g^–1^ DW, phosphorous = 2.98 mg g^–1^ DW, potassium = 11.97 mg g^–1^ DW, and calcium = 16.8 mg g^–1^ DW. The extracts were freshly prepared before their application. Distilled water was sprayed on control plants in both applications. MLE was sprayed to a pot-grown wheat twice after 25 and 35 days of sowing date (50 ml per plant) after the establishment of drought stress. Tween-20 surfactant was used in a foliar spray (0.1% v/v).

### Harvesting and Measurement of Growth Attributes

Harvest was done after 45 days of planting. Root and shoot fresh weights (FWs) and lengths were measured immediately after harvest at the experimental site. Fresh samples were stored at -30°C in a biomedical refrigerator for fresh analysis. Three samples per treatment were oven-dried (65°C) for 3 days to determine their dry weights (DWs) and ionic content analysis using the acid digestion method.

### Photosynthetic Pigments

The chlorophyll contents of wheat leaves were determined as described by Dere et al. (1998). Notably, 0.2 g of leaves (randomly collected) were extracted in 10 ml methanol (96%) using a homogenizer at 1,000 rpm for 1 min, then filtered, and centrifuged for 10 min at 2,500 rpm. The supernatant was separated and used to determine chlorophyll contents at 666 (chlorophyll a), 653 (chlorophyll b), and 470 nm (total carotenoids) wavelengths using a spectrophotometer (Model SM1200; Randolph, NJ, United States).

### Measurements of Stomatal Conductance (gs)

Stomatal conductance (gs) of fully developed leaves (three plants per treatment) was measured by putting them in a portable infrared gas analyzer chamber (Analytical Development Company, Hoddeson, United Kingdom). The measurements were made 6 days after the first MLE foliar spray.

### Leaf Biochemical Analysis

Fully expanded leaves from each replicate were taken, wrapped in aluminum foils, immersed in liquid nitrogen, and transferred into plastic zipper bags. These samples were stored at –80°C for further analysis. Following, biochemical analysis was performed using a spectrophotometer (Model SM1200; Randolph, NJ, United States).

To determine osmolytes as sugars and non-enzymatic antioxidants, 50 mg of dried leaves were homogenized in 10 ml of 80% ethanol and filtered followed by the re-extraction in 10 ml ethanol, and a 20 ml of the final volume was maintained. This obtained solution was used to evaluate flavonoids (Lewis et al., 1999), soluble sugars (Dubois et al., 1956), proteins (Bradford, 1976), and proline (Bates et al., 1973) contents. Glycine betaine (GB) was assessed by following the method of Holmström et al. (2000).

### Mineral Content

For the determination of P contents in wheat molybdate/ascorbic acid, the blue technique was used, and nitrate contents were assessed by Kowalenko and Lowe (1973) method using a spectrophotometer (Model SM1200; Randolph, NJ, United States). K concentration was determined using a flame photometer. Ca and Na ions were evaluated by Atomic Absorption Spectrum (AAS; Shimadzu instruments, Inc., Spectra AA-220, Kyoto, Japan).

### Statistical Analysis

Statistical analysis of data was performed using a *post-hoc* test, which was performed to measure specific differences between treatments using the Duncan’s Multiple Range Test (DMRT) in a completely randomized block design. The significant diffidences between treatment means were determined using analysis of variance and mean separation at a 5% significance level (*p* ≤ 0.05). In addition, the Pearson correlation of different wheat attributes under drought stress and well-watered conditions was performed. Logarithmic data transformation to obtain near-normal distribution was implemented before analysis, where required.

## Results

### Length, Fresh Weight, and Dry Weight of Roots and Shoot

**FIGURE 1:**
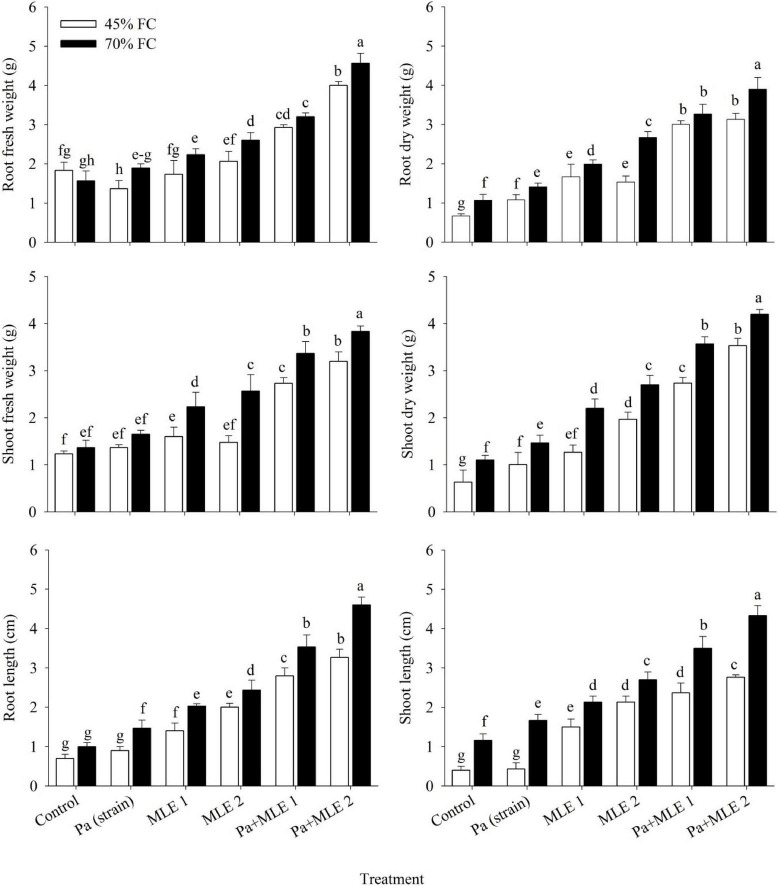
Impact of sole and combined applications of Pa and moringa leaf extract (MLE) on different growth attributes of wheat plants under 45 FC and 70 FC irrigation levels. Different bars represent the mean values of three replicates. Error bars represent standard error (SE). Different letters on bars indicate significant difference at *p* ≤ 0.05 [70 FC = well-irrigated, 45 FC = drought stress condition; C = control, Pa (strain) = *Pseudomonas aeruginosa* inoculated plants, MLE 1 = foliar-applied MLE at 1:15, and MLE 2 = foliar-applied MLE at 1:30]. Means with the same letters within the column are not significantly different.

All applied treatments had a significant positive effect on root and shoot FWs, DWs, and lengths under control and drought stress conditions (Figure 1). In this regard, Pa + MLE 1 and Pa + MLE 2 were proved significantly efficient amendments in increasing root FW and shoot FW of wheat plants during drought conditions. Furthermore, MLE 2 enhanced root DW and shoot DW at 45 FC compared with control (70 FC). The treatments MLE 2, Pa + MLE 1, and Pa + MLE 2 had a more significant effect on root length and shoot length as compared with the control under drought stress. A significant difference was observed between the given amendments and control plants which signify their effectiveness in drought stress environments.

### Photosynthetic Pigments

**FIGURE 2:**
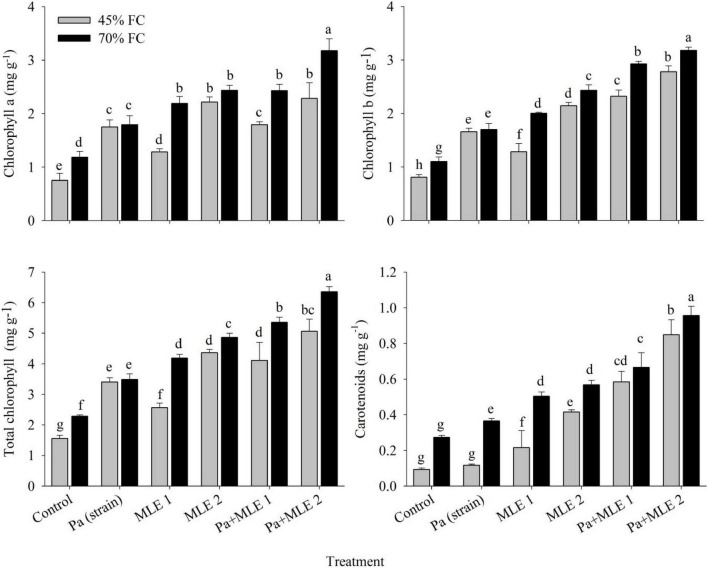
Impact of sole and combined applications of Pa and MLE on different photosynthetic pigments of wheat plants under 45 FC and 70 FC irrigation levels. Different bars represent the mean values of three replicates. Error bars represent SE. Different letters on bars indicate significant difference at *p* ≤ 0.05 [70 FC = well-irrigated, 45 FC = drought stress condition; C = control, Pa (strain) = *P. aeruginosa* inoculated plants, MLE 1 = foliar-applied MLE at 1:15, and MLE 2 = foliar-applied MLE at 1:30]. Means with the same letters within the column are not significantly different.

All photosynthetic pigments such as chlorophyll a, chlorophyll b, total chlorophyll, and carotenoids were significantly affected when subjected to drought stress conditions (45 FC) as compared with well-irrigated conditions (70 FC) (Figure 2). All applied amendments significantly improved plant photosynthetic pigments over the control. Pa + MLE 1 and Pa + MLE 2 treatments were remained efficient in increasing chlorophyll a and chlorophyll b at 45 FC over control-treated wheat plants (70 FC). Furthermore, MLE 2 differed significantly better for the enhancement of total chlorophyll contents at 45 FC than 70 FC. Treatments MLE 2, Pa + MLE 1, and Pa + MLE 2 were significantly different for wheat carotenoid contents under drought stress as compared with the control. A significant difference was observed in MLE at both concentrations and Pa for all studied parameters in all treatment levels. The above results signify the value of given amendments in enhancing the efficiency of water uptake and use by the grown plants.

### Flavonoids, Phenolics, Total Soluble Sugars, Total Soluble Proteins, and Stomatal Conductance

**FIGURE 3:**
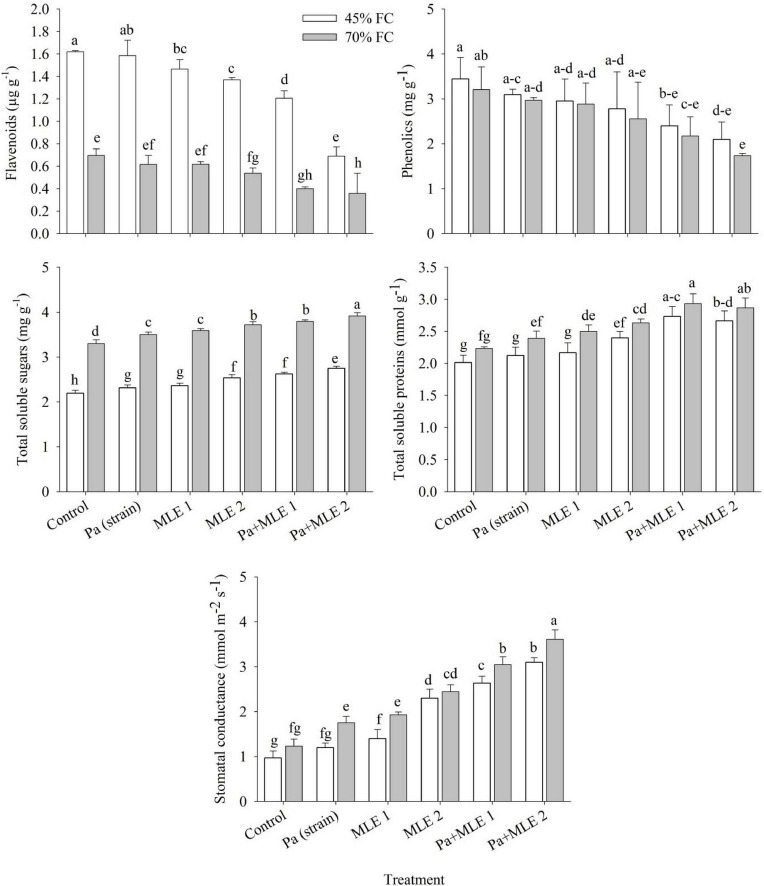
Impact of sole and combined applications of Pa and MLE on flavonoids, phenolics, total soluble sugars, total soluble proteins, and stomatal conductance of wheat plants under 45 FC and 70 FC irrigation levels. Different bars represent the mean values of three replicates. Error bars represent SE. Different letters on bars indicate significant difference at *p* ≤ 0.05 [70 FC = well-irrigated, 45 FC = drought stress condition; C = control, Pa (strain) = *P. aeruginosa* inoculated plants, MLE 1 = foliar-applied MLE at 1:15, MLE 2 = foliar-applied MLE at 1:30, and S.C. = stomatal conductance]. Means with the same letters within the column are not significantly different.

Flavonoids, phenolics, total soluble sugars, total soluble proteins, and stomatal conductance of wheat plants were significantly affected in drought stress (45 FC) compared with well-watered plants (70 FC) (Figure 3). Both sole and combined applications of Pa and MLE significantly increased plant total soluble sugars, total soluble proteins, and stomatal conductance over the control. Pa + MLE 1 and Pa + MLE 2 treatments were remained efficient in decreasing flavonoids and phenolics at 45 FC over 70 FC. Moreover, MLE 2 differed significantly better than MLE 1 and Pa either in sole or combined applications at 45 FC than 70 FC, while, MLE 2, Pa + MLE 1, and Pa + MLE 2 were significantly efficient in decreasing flavonoids and phenolics of wheat plants under drought stress as compared with the control. These results prove the effectiveness of these amendments in increasing the water uptake efficiency of wheat plants.

### Oxidative Stress Indicators, Enzymatic Antioxidants, and Ionic Contents

Ionic nutrient contents increased significantly in plants that were irrigated at 70 FC compared with plants irrigated with water to reach only 45 FC (Table 1). However, in contrast, different oxidative stress indicators such as sodium, proline, GB, electrolyte leakage (EL), malondialdehyde (MDA), and hydrogen peroxide (H_2_O_2_) increased significantly in plants subjected to 45 FC water conditions. Furthermore, antioxidant enzyme [superoxide dismutase (SOD) and peroxide (POD)] activities were increased in drought stress.

**TABLE 1:** Oxidative stress indicators, enzymatic antioxidants, and ionic contents of wheat grown in well-watered and drought stress conditions.

Treatment	Proline (μg g^–1^ FW)	GB (μmol g^–1^)	EL (%)	MDA (μmol g^–1^ FW)	H_2_O_2_ (μmol g^–1^ FW)	SOD (U g^–1^ FW)	POD (U g^–1^ FW)	Sodium (mg g^–1^ DW)	Calcium (mg g^–1^ DW)	Potassium (mg g^–1^ DW)	Phosphate (mg g^–1^ DW)	Nitrate (mg g^–1^ DW)
	**45% FC**											
Control	3.40 ± 0.09a	2.03 ± 0.29a	30.33 ± 2.52a	1.85 ± 0.07a	3.26 ± 0.53a	1.92 ± 0.35a	6.15 ± 1.06a	22.67 ± 1.53a	8.33 ± 0.76i	3.17 ± 0.29ef	0.02 ± 0.0h	3.18 ± 0.02e
Pa (strain)	3.27 ± 0.08b	1.83 ± 1.54ab	27.67 ± 2.08ab	1.13 ± 0.24bc	2.96 ± 0.48a-c	1.76 ± 0.13ab	4.06 ± 0.33b-d	21.67 ± 0.58ab	10.17 ± 0.76hi	4.33 ± 0.58e	0.02 ± 0.0f-h	3.27 ± 0.02e
MLE 1	3.13 ± 0.04c	1.78 ± 0.05ab	25.67 ± 1.53b	1.22 ± 0.64b	3.09 ± 0.29ab	1.77 ± 0.15ab	4.23 ± 1.03bc	20.00 ± 2.65b	12.83 ± 0.76fg	4.33 ± 0.76e	0.02 ± 0.0gh	3.25 ± 0.05e
MLE 2	2.89 ± 0.06d	1.50 ± 0.28ab	26.33 ± 0.58b	1.11 ± 0.13bc	2.68 ± 0.59a-d	1.62 ± 0.39a-c	3.79 ± 0.41b-d	16.33 ± 0.58cd	13.67 ± 1.76f	3.17 ± 0.76ef	0.03 ± 0.01e-g	3.43 ± 0.06d
Pa + MLE 1	2.77 ± 0.05de	1.22 ± 0.43a-c	20.67 ± 2.52cd	1.09 ± 0.02bc	2.53 ± 0.27a-d	1.28 ± 0.32a-d	3.05 ± 0.57de	14.67 ± 0.58de	17.67 ± 1.61cd	2.50 ± 1.0f	0.04 ± 0.01c-e	3.63 ± 0.06c
Pa + MLE 2	2.63 ± 0.09fg	1.10 ± 0.01a-c	17.67 ± 1.53de	0.78 ± 0.14b-d	2.27 ± 0.09cd	0.73 ± 0.10c-e	2.47 ± 0.56ef	13.33 ± 1.15ef	21.17 ± 0.76b	1.97 ± 0.06f	0.04 ± 0.0bc	3.87 ± 0.06b
	**70% FC**											
Control	2.88 ± 0.09d	1.20 ± 0.04a-c	21.67 ± 2.08c	0.85 ± 0.23b-d	2.47 ± 0.27b-d	1.16 ± 1.37a-d	4.62 ± 0.55b	21.17 ± 1.26ab	11.00 ± 1.0gh	4.33 ± 1.04e	0.03 ± 0.0fg	3.23 ± 0.03e
Pa (strain)	2.75 ± 0.08ef	0.99 ± 0.28bc	19.67 ± 1.53cd	0.70 ± 0.54b-d	2.23 ± 0.22cd	0.83 ± 0.07c-e	3.08 ± 0.51de	20.00 ± 0.50b	11.67 ± 1.53gh	4.67 ± 1.53e	0.03 ± 0.0d-f	3.40 ± 0.02d
MLE 1	2.59 ± 0.07gh	1.12 ± 0.33a-c	17.00 ± 1.0d-f	0.79 ± 0.12b-d	2.32 ± 0.16cd	0.96 ± 0.27b-e	3.54 ± 0.11c-e	17.00 ± 1.0c	14.33 ± 0.58ef	6.83 ± 0.76d	0.04 ± 0.0c-e	3.48 ± 0.03d
MLE 2	2.48 ± 0.05hi	0.93 ± 0.28bc	18.00 ± 2.65c-e	0.61 ± 0.07cd	2.19 ± 0.13d	0.71 ± 0.46c-e	2.67 ± 0.09ef	14.33 ± 0.58de	16.17 ± 1.04de	8.50 ± 0.5c	0.04 ± 0.0b-d	3.63 ± 0.06c
Pa + MLE 1	2.37 ± 0.05ij	0.35 ± 0.2c	15.33 ± 1.53ef	0.55 ± 0.15d	2.14 ± 0.14d	0.43 ± 0.44de	2.48 ± 0.29ef	12.67 ± 0.58ef	19.50 ± 1.32bc	10.00 ± 0.5b	0.05 ± 0.01b	3.87 ± 0.0b
Pa + MLE 2	2.26 ± 0.07j	0.26 ± 0.06c	13.67 ± 3.21f	0.36 ± 0.22d	2.01 ± 0.83d	0.15 ± 0.06e	1.97 ± 0.48f	11.33 ± 1.1f	23.33 ± 0.76a	12.67 ± 1.2a	0.07 ± 0.01a	4.21 ± 0.1b

*Means with the same letters within the column are not significantly different.*

All additives had significant positive improvements in ameliorating drought effects in wheat. These amendments were more detectable when the dose of MLE foliar application was increased. Pa and MLE decreased sodium, proline, GB, EL, MDA, H_2_O_2_, and SOD and POD contents significantly. Also, they increased calcium, potassium, phosphate, and nitrate concentrations in plant tissues. It is worth mentioning in this study that sole and combined applications of either MLE or Pa were better in decreasing the oxidative stress indicators and increasing nutrient contents of wheat, and this signifies the success of these ameliorating treatments for drought stress. Pa + MLE 1 and Pa + MLE 2 recorded the least oxidative stress indicators and enzymatic antioxidant activities during more nutrient assimilation in plants.

All values are the means of three replicates ± SD. Different labels represent significant different alphabets using the least significant difference (LSD) test. [70 FC = well-irrigated conditions, 45 FC = drought stress conditions; C = control, Pa (strain) = *P. aeruginosa* inoculated plants, MLE 1 = foliar applied MLE at 1:15, and MLE 2 = foliar applied MLE at 1:30] [GB = glycine betaine, EL = electrolyte leakage, MDA = malondialdehyde, H_2_O_2_ = hydrogen peroxide, SOD = superoxide dismutase, and POD = peroxide].

### Pearson Correlation

**FIGURE 4:**
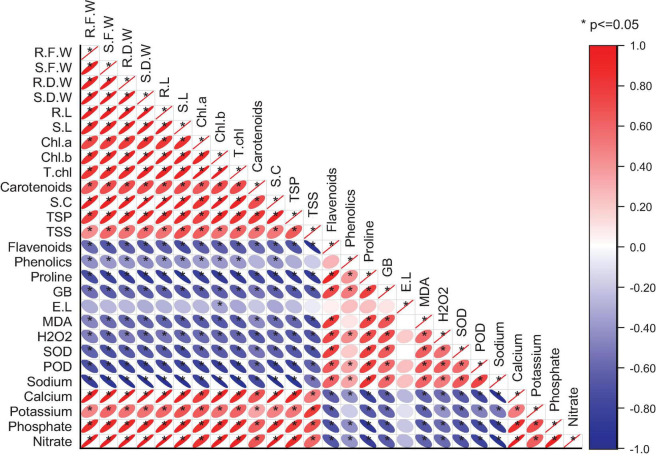
Pearson correlation of different wheat attributes under drought stress and well-watered conditions.

A significant positive correlation exists between plant morphological attributes and photosynthetic pigments (Figure 4). This might, in turn, improve plant vigor and consequently improved plant growth. Also, the total soluble sugars, total soluble proteins, stomatal conductance, and ionic contents were increased with the given amendments. However, oxidative stress indicators, enzymatic antioxidants, flavonoids, and phenolics increased under drought stress conditions recorded significant negative correlations with plant growth parameters.

## Discussion

This study evaluates the effect of MLE (MLE 1 = 1:20 v/v and MLE 2 = 1:30 v/v) and PGPR strain (Pa) on drought stress tolerance ability of wheat (Anaj 17 genotype) under two irrigation regimes (45 FC and 70 FC). The use of organic or biofertilizers as a global initiative instead of costly chemical fertilizers is addressed by several researchers (Talaei et al., 2014; El-Naggar et al., 2015; El-Serafy, 2018; Rahimi et al., 2019). Plant-derived biostimulants that trigger abiotic stress tolerance to ameliorate stress-induced yield reduction involve many different mechanisms, and most of these involve phytohormones and upregulated antioxidant defense systems (Bulgari et al., 2017; Sadasivam et al., 2017).

The highest dry matter contents in both root and shoot were obtained with the combined application of Pa + MLE 2 mixture that agrees with previous findings describing that soil beneficial bacteria can promote plant growth in abiotic stress conditions. Durán et al. (2016) had similar results on lettuce inoculated with *Bacillus* sp. and *Klebsiella* sp. and concluded that inoculated plants have more DW than non-inoculated ones. In this sense, Glick et al. (2007) said that this increase in the dry matter might be the enzyme 1-aminocyclopropane-1-carboxylate (ACC) deaminase enzyme activity of bacteria to lower the stress-induced ethylene levels, leaving α-ketobutyrate and NH4^+^ available to the plants. Moreover, according to the study by Sánchez López (2011), auxins, i.e., indole-3-acetic acid (IAA), have a stimulatory effect on secondary root development that contributes to the total biomass of the plant by increasing nutrient and water absorption in moisture deficit conditions. The results from this study are in accordance with those claimed by Mishra et al. (2012) that an increase in auxin concentration stimulates adventitious root formation and thus increases root surface area and total plant biomass, proclaiming a morphological strategy of plants to tolerate drought with the help of bacterial strains.

Photosynthetic pigment contents are considered a valuable physiological indicator for evaluating the damage caused by stress intensity. The wheat genotype in this study had more chlorophyll contents when supplemented with Pa and MLE with the highest value recorded in their combined treatment at both water regimes. This alteration is directly related to plant macronutrient contents such as nitrogen. The reactive oxygen species (ROS) produced during abiotic stress cause the reduction of chlorophyll contents by damaging photosynthetic machinery (Manivannan et al., 2007). Similar to our results, Ahmadi et al. (2013) also found a significant increase in chlorophyll contents by inoculating wheat with different bacterial strains (*Pseudomonas* sp. *and Azospirillum* sp.).

This study indicates that the increase in mineral nutrient contents of wheat is due to the use of biostimulants that enhanced drought stress tolerance. The inoculation with *P. aeruginosa* and MLE applied separately and in combination increased levels of sodium, calcium, potassium, phosphate, and nitrate in wheat submitted to drought. This might be due to the nutrient solubilization by added microorganisms due to the production of ACC deaminase (Mutumba et al., 2018). In addition, Römheld and Kirkby (2010) considered potassium as a key element in reducing water stress by adjusting osmoregulation in cells as a compatible solute. Zulfiqar et al. (2020) reported enhancement in soil nutrient availability and improvement in fruit quality induced by plant-derived biostimulants in stress conditions. The increase in plant nutritional status and vigor is due to improved soil microbial activity (Colla et al., 2015) and the presence of some chelating agents that enhance nutrient solubility in soil (Abou Chehade et al., 2018).

Induced tolerance by plant growth regulators is triggered by external stress factors and enhancement in plant sterols by reducing EL and regulating membrane stability (Lucini et al., 2015), thus providing an antioxidant defense system. Results of this experimental study indicate that Pa and MLE significantly improve growth, biochemical, antioxidant, and ionic attributes of wheat. Rhizobacteria potentially increase plant vigor by increasing atmospheric nitrogen fixation ability of roots and mineral absorption.

In accordance with our findings, previous studies also validate drought stress effects that consequently increase ROS that activate plant enzymatic and non-enzymatic antioxidant systems in order to balance the cellular redox state (Zulfiqar et al., 2020). Kumar et al. (2019) reported seed priming that involves plant growth regulators inducing sugar-triggered plant immunity (called “sweet immunity”—immunity by sugar compounds) having a positive priming effect on antioxidant systems. Results of our study validate that the antioxidant contents were improved using MLEs as a bioactive compound. Furthermore, a study by Rashid et al. (2018) confirmed the presence of a primary growth regulator, zeatin (a cytokinin derivative) that provided abiotic stress tolerance. Moringa extract constitutes considerable quantities of secondary metabolites, antioxidants, and osmoprotectants (Rehman et al., 2015). MLE’s growth-promoting and abiotic stress tolerance inducing effects justify its use as a potential alternative to synthetic chemical fertilizers for improving wheat productivity (Nasir et al., 2016; Brockman and Brennan, 2017; Merwad, 2018; Younis et al., 2018). MLE aqueous solutions are very easy to make, cost-effective, and environment-friendly and can be used by the farmers effectively for increasing their crop yields. This study clearly reveals that all studied growth, biochemical, ionic, and antioxidant constituents of wheat plants were improved by using either of the two given amendments. Pa and MLE reduced all investigated oxidants and oxidative stress indicators. It is worth mentioning in this study that these supplementary amendments might antagonize each other’s effects to some extent in their combined applications, as their sole applications had lowered physiological, biochemical, and ionic attributes. Thus, constitutive effects, i.e., Pa and MLE, at both concentration levels were more elaborated, thus inducing drought tolerance in wheat.

## Conclusion

The application of both nitrogen-fixing bacteria (Pa strain) and foliar sprayed MLE either in sole or combined treatments can alleviate nutrient and drought stress effects. Nevertheless, the combined application of Pa and MLE 2 (Pa + MLE 2) can efficiently improve wheat growth attributes, photosynthetic pigment contents, and nutrient uptake under drought stress. The combined supplementation of Pa and MLE had more significant positive effects compared with their sole applications. Pa + MLE 2 was also efficient in decreasing oxidative stress indicators and enzymatic antioxidants of wheat under drought stress. There is a need for more investigations at field levels by considering the effect of other environmental constraints to effectively validate these findings.

## Data Availability Statement

The original contributions presented in the study are included in the article/supplementary material, further inquiries can be directed to the corresponding author/s.

## Author Contributions

IL, SA-D, LA-A, RH, KAM, NM, SA, MA, AA, PP, KM, and TG: researching and writing. AA, SA, and LA-A: writing. All authors contributed to the article and approved the submitted version.

## Conflict of Interest

The authors declare that the research was conducted in the absence of any commercial or financial relationships that could be construed as a potential conflict of interest. The reviewer FZ declared a shared affiliation with one of the author, RH, to the handling editor at the time of the review.

## Publisher’s Note

All claims expressed in this article are solely those of the authors and do not necessarily represent those of their affiliated organizations, or those of the publisher, the editors and the reviewers. Any product that may be evaluated in this article, or claim that may be made by its manufacturer, is not guaranteed or endorsed by the publisher.
